# Editorial: Community series in tumor microenvironment and metabolic reprogramming in cancer: volume II

**DOI:** 10.3389/fimmu.2026.1898013

**Published:** 2026-06-12

**Authors:** Yaodong Sang, Huanyu Gao, Jingsi Li, Asif Raza, Houjuan Zhu, Xiaoling Cui, Yiju Wei, Hao Chen, Wei Chong

**Affiliations:** 1 Department of Gastrointestinal Surgery, Shandong Provincial Hospital Affiliated to Shandong First Medical University, Jinan, China; 2 Shandong Provincial Laboratory of Translational Medicine Engineering for Digestive Tumors, Shandong Provincial Hospital, Jinan, China; 3 Medical Science and Technology Innovation Center, Shandong First Medical University & Shandong Academy of Medical Sciences, Jinan, China; 4 Shandong Provincial Hospital Affiliated to Shandong First Medical University, Jinan, China; 5 Department of Pharmacology, Penn State Cancer Institute, The Pennsylvania State University College of Medicine, Hershey, PA, United States; 6 Institute of Materials Research and Engineering (IMRE), Agency for Science, Technology and Research (ASTAR), Singapore, Singapore; 7 School of Life Science, Shandong First Medical University & Shandong Academy of Medical Sciences, Jinan, Shandong, China; 8 Clinical Research Center of Shandong University, Clinical Epidemiology Unit, Qilu Hospital of Shandong University, Jinan, China

**Keywords:** cancer, immunosuppression, metabolic reprogramming, TME (tumor microenvironment), tumor

The tumor microenvironment (TME), composed of tumor cells, various surrounding cellular components, and the extracellular matrix (ECM), plays a critical role in tumor initiation, progression, and metastasis (1). Metabolic reprogramming is a hallmark of cancer. The hypoxic, nutrient-deprived, and acidic TME drives tumor metabolic reprogramming; conversely, tumor metabolites reshape the TME and suppress antitumor immunity (2). This editorial synthesizes insights from 19 recent studies that elucidate the complex and interdependent regulatory network linking the tumor microenvironment, metabolic reprogramming, and tumor progression. These findings highlight the key roles of this network in tumorigenesis, immune evasion, and therapeutic resistance, providing new perspectives and a theoretical basis for metabolism-targeted cancer therapies.

Metabolic reprogramming in tumor cells actively shapes an immunosuppressive microenvironment (3). Multiple studies have revealed distinct metabolic characteristics across different tumor types. A recent review by Shan and Liu systematically elucidated the regulatory mechanisms of glycolytic reprogramming in gastric cancer, ranging from non-coding RNAs to epigenetic modifications, providing insights for targeting the Warburg effect. Similarly, Liu et al. pointed out that abnormal hyperactivity of nucleotide metabolism is a key factor supporting the rapid proliferation and chemotherapy resistance of pancreatic cancer, in which enzymes such as RRM2 are highly promising therapeutic targets. The complexity of metabolic reprogramming is equally prominent in liver cancer. Yang et al. elucidated how hepatocellular carcinoma reprograms glucose, glutamine, and lipid metabolism to interact with stellate cells, fibroblasts, and immune cells in the microenvironment, collectively promoting tumor progression. Similarly, in cholangiocarcinoma, Xue et al. further focused on the “immune-metabolic” dual regulation, proposing that targeting lactate, adenosine, and bile acid signaling axes holds promise for converting “cold tumors” into “hot tumors” and sensitizing immunotherapy. Finally, Su et al. highlighted the universality and challenges of metabolic targeted therapy from another perspective, pointing out that activation of the serine synthesis pathway supports proliferation, maintains redox homeostasis, and shapes the immunosuppressive microenvironment in multiple tumor types. However, targeting this pathway requires consideration of tumor subtype and microenvironment specificity. Thus, although different tumors exhibit distinct metabolic characteristics-a notion supported by comparative metabolomic studies across cancer types (4)-targeting these core metabolic pathways has become a consensus in cancer research.

Metabolic reprogramming in tumors is closely linked to the functional state of immune cells within the TME. Zhou et al. reported a case of a patient with renal cell carcinoma who successfully underwent delayed cytoreductive nephrectomy following treatment with axitinib combined with toripalimab, demonstrating the clinical value of remodeling the metabolic and immune microenvironment. In osteosarcoma, Wang and Shi detailed how tumor-derived exosomes induce macrophage polarization toward the M2 phenotype and suppress T cell function by carrying functional molecules such as TGF-β, thereby constructing an immunosuppressive microenvironment. Ivanova et al. found that autophagy-related proteins are highly expressed in both stromal cells and tumor cells in colorectal cancer, and are associated with tumor budding and poor prognosis, suggesting active metabolic crosstalk at the invasive front.In addition, Ning and Huang focused on the ‘tryptophan-kynurenine-AhR’ axis in head and neck squamous cell carcinoma, pointing out that this pathway forms a spatially confined immunometabolic niche within the tumor microenvironment, which is a major cause of T cell dysfunction. Furthermore, Jiang et al. systematically reviewed the phenotypic plasticity and functional reprogramming of innate and adaptive immune cells in non-small cell lung cancer, emphasizing the profound impact of immune cell heterogeneity on immunotherapy response and resistance. These studies indicate that the functional state of immune cells determines the immune phenotype of the tumor microenvironment (TME), and accumulating evidence across tumor types has consolidated this immunometabolic paradigm (5), thereby influencing patient treatment outcomes.

In a pan-cancer analysis, Zhang et al. identified that KIF20A is highly expressed in multiple tumor types and is associated with poor prognosis and immune cell infiltration. Their experimental validation supports its potential as a therapeutic target. Similarly, Lyu et al. revealed the oncogenic role and prognostic value of SLC2A3 in clear cell renal cell carcinoma, and further validated its regulation of cell proliferation and migration through *in vitro* experiments. Furthermore, a pan-cancer analysis by Wang et al. demonstrated that high expression of IGSF8 is associated with poor prognosis in multiple tumor types and, notably, is positively correlated with CD276 expression, suggesting its potential as a novel immune checkpoint. In addition, some studies have also revealed unconventional regulatory pathways. This focus on immune-related targets has gradually expanded to include non-canonical pathways that bridge metabolic regulation and innate immunity. Ni et al. revealed that in gastric cancer, the complement C5a-C5aR pathway promotes iron transport to tumor cells by inducing M2 polarization of macrophages and upregulating LCN2 via endoplasmic reticulum stress, thereby supporting tumor progression, providing a novel perspective on the regulation of tumor metabolism by the complement system.Meanwhile, using spatially resolved imaging mass cytometry, Feng et al. identified a fibroblast subset associated with abnormal angiogenesis and poor prognosis at the invasive front of bladder cancer, providing new insights for spatially targeted therapy of the tumor microenvironment (TME).

The roles of gaseous signaling molecules and systemic factors in TME remodeling are receiving increasing attention. Beyond the canonical gasotransmitters, emerging evidence indicates that these gaseous molecules orchestrate a complex network of regulation within the TME (6). Ballerini et al. comprehensively summarized the dual functions of nitric oxide(NO), carbon monoxide(CO), and hydrogen sulfide(H2S)in tumor-immune interactions, pointing out that these small gaseous molecules modulate redox balance and metabolic signaling, and that their precise regulation may hold the key to next-generation immunocombination therapies. Yang et al.through bibliometric analysis, systematically reviewed two decades of research hotspots in the fields of TME and metabolic reprogramming, predicting that lipid metabolism, non-coding RNAs, exosomes, and immunotherapy will continue to be future directions in this area, providing macro-level guidance for subsequent studies.

Emerging therapeutic and predictive approaches are driving progress in cancer treatment (7). Qu et al. achieved active targeted delivery of resveratrol to liver cancer cells using nanomaterials, providing an excellent example of the application of active ingredients from traditional Chinese medicine. Zhang et al. successfully predicted pathological complete response to neoadjuvant chemotherapy in HER2-negative breast cancer patients using an artificial neural network based on five immune-related genes, demonstrating the potential of multi-omics data to guide personalized therapy.

In conclusion, these cutting-edge studies have profoundly revealed the complex crosstalk between the tumor microenvironment (TME) and metabolic reprogramming, providing unique insights ranging from molecular mechanisms and biomarkers to innovative therapies. Future cancer treatment will integrate multi-omics analysis, spatial biology technologies, and combined targeted metabolic-immune therapies, representing a key pathway toward more effective and personalized cancer therapy.
